# Beta-Alanine Supplementation Improved 10-km Running Time Trial in Physically Active Adults

**DOI:** 10.3389/fphys.2018.01105

**Published:** 2018-08-08

**Authors:** Jeferson O. Santana, Marcelo C. de Freitas, Diana M. dos Santos, Fabrício E. Rossi, Fabio S. Lira, José C. Rosa-Neto, Erico C. Caperuto

**Affiliations:** ^1^Department of Physical Education, University São Judas Tadeu, São Paulo, Brazil; ^2^Skeletal Muscle Assessment Laboratory, Department of Physical Education, School of Technology and Sciences, São Paulo State University, Presidente Prudente, Brazil; ^3^Immunometabolism of Skeletal Muscle and Exercise Research Group, Department of Physical Education, Federal University of Piauí, Teresina, Brazil; ^4^Exercise and Immunometabolism Research Group, Department of Physical Education, São Paulo State University, Presidente Prudente, Brazil; ^5^Biomedical Sciences Institute, São Paulo University, São Paulo, Brazil

**Keywords:** sport nutrition, endurance training, performance, supplementation, running exercise

## Abstract

The purpose of this study was to investigate the effects of β-alanine supplementation on a 10 km running time trial and lactate concentration in physically active adults. Sixteen healthy subjects were divided randomly into two groups: β-alanine (*n* = 8) and placebo group (*n* = 8). The experimental group ingested 5 g/day of β-alanine plus 1 g of resistant starch, and control group ingested 6 g of resistant starch, both for 23 days. Time to complete a 10-km running time trial and lactate concentration following the test were assessed at baseline and post 23 days. The running training program was performed three times per week on non-consecutive days (day 1: running 7 km; day 2: six sprints of 500 m at maximum speed with 2 min of recovery; day 3: running 12 km). The time to complete a 10-km running time trial decreased significantly only for the β-alanine group (Pre = 3441 ± 326.7, Post = 3209 ± 270.5 s, *p* < 0.05). When analyzing the delta (Time post minus Time at baseline value) there was a statistically significant difference between the β-alanine vs placebo group (-168.8 ± 156.6 vs. -53.60 ± 78.81 s, *p* = 0.007), respectively. In addition, the β-alanine group presented lower blood lactate concentration after the 10-km test (β-alanine: Pre = 8.45 ± 1.94 vs. Post = 6.95 ± 2.44 mmol/L; Placebo: Pre = 8.7 ± 3.0 vs. Post = 10.8 ± 2.5 mmol/L, *p* = 0.03). In conclusion, β-alanine supplementation improved the 10-km running time trial and reduced lactate concentration in physically active adults.

## Introduction

Beta-alanine (β-alanine) is a non-proteinogenic amino acid that combined with histidine can result in a dipeptide called carnosine, formed through an ATP-dependent reaction inside skeletal muscle mass (Tiedje et al., 2010). Daily doses of 4.8–6.4 g of β-alanine over 23 or 28 days can elevate human muscle carnosine content after supplementation (Harris et al., 2006; Bex et al., 2014). However, equimolar carnosine intake does not elevate muscle carnosine more than β-alanine alone (Derave et al., 2007) and the physiological roles of intramuscular carnosine during exercise suggest β-alanine supplementation as a great tool to enhance exercise performance.

Previous studies have shown that β-alanine increased the intramuscular buffering of hydrogen ions (H^+^), delaying the acidosis induced during high-intensity exercise (Saunders et al., 2017). Another physiological role of carnosine that may explain these ergogenic effects is to increase calcium sensitivity in muscle fibers and the amount of work performed (Dutka and Lamb, 2004; Dutka et al., 2012). Therefore, the increase in carnosine content could attenuate fatigue not only through its buffering capacities, but also its ability to improve myofibrillar Ca^2+^ sensitivity (Sale et al., 2010).

Some investigations analyzed the effects of β-alanine supplementation on performance in different exercise types and program structures. Meta analyses studies have demonstrated that the effects of β-alanine supplementation on performance are dependent on exercise duration and intensity. Saunders et al. (2017) observed that exercise lasting from 0.5 to 10 min shows the best results, while brief exercise (<0.5 min) does not present any improvement in performance. Hobson et al. (2012) also demonstrated that β-alanine is most effective during exercise of 60–240 s in duration, suggesting that this is due to the fact that maximum H^+^ accumulation occurs after approximately 4 min of high-intensity exercise (Osnes and Hermansen, 1972). However, the authors related that few studies have examined long-duration continuous exercises, and the majority of studies used an incremental protocol. In addition, the latest position stand on β-alanine reported that more research is necessary to determine the effects of β-alanine on endurance performance beyond 25 min in duration (Trexler et al., 2015).

Furthermore, the majority of investigations of β-alanine in the literature used a cycle ergometer; however, few studies have analyzed the influence of β-alanine supplementation on long-distance running performance. Ducker et al. (2013) found that β-alanine supplementation (4 weeks of 80 mg⋅kg-day) improves 800 m track running performance in male recreational club runners. On the other hand, Smith et al. (2012) did not find improvement in 40 min of treadmill running in women after 28 days of β-alanine supplementation (2 × 800 mg tablets, 3× daily). Hoffman et al. (2015) examined soldiers from an elite combat unit after 30 days of β-alanine supplementation (6 g per day) and did not find improvement in 2.5 km running, 30-m repeated sprint, or the 1-min sprint test, although they observed an increase in 50-m casualty carrying. Therefore, whether β-alanine supplementation influences 10-km running performance is currently unknown.

Although long-distance running relies mainly on aerobic energy metabolism, higher lactate concentrations have been associated with lower speed in prolonged running (Sjodin and Jacobs, 1981; Fohrenbach et al., 1987; Tanaka, 1990). Previous studies have demonstrated that β-alanine supplementation can reduce blood lactate accumulation during an incremental running test (Jordan et al., 2010; Ghiasvand et al., 2012). Glenn et al. (2015) showed that β-alanine supplementation (four doses/day with 800 mg beta-alanine + 8 g dextrose) for 28 days reduced 24% of blood lactate concentrations after cycling test to exhaustion (equivalent to 120% maximal oxygen uptake) in female cyclists. Thus, improvements in prolonged running performance with β-alanine may be plausible, mainly due to the effects of β-alanine on lowering lactate accumulation during exercise.

Therefore, the aim of this study was to investigate the effects of β-alanine supplementation on a 10 km running time trial and lactate concentration in physically active adults.

## Materials and Methods

### Experimental Approach to the Problem

**FIGURE 1:**
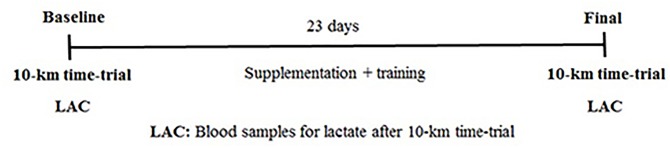
Experimental design.

This study used a randomized, double-blind, crossover design (**Figure 1**). The participants were divided randomly into: β-alanine group and placebo group. All subjects performed the same running training protocol during the study. The subjects completed 10-km running tests and blood lactate concentration was measured after the 10-km tests before and after 23 days of supplementation.

### Subjects

Sixteen healthy men (**Table 1**) were recruited for this study. As inclusion criteria were defined: (i) at least 6 months of running experience, (ii) personal best time in 10-km between 55 and 65 min; and (iii) performing at least two to three training sessions per week. Subjects were instructed not to use any supplements or ergogenic substance during the experimental protocol. We excluded subjects that were absent in 25% of the training sessions, and/or did not take the supplement as prescribed and/or changed their normal diet. Subjects with pre-existing illnesses that would impair training or those without a medical approval form were also excluded. All experimental procedures were approved by the University Ethical Committee under protocol number CAAE: 38414814.3.0000.0089. Informed consent was obtained from all individual participants included in the study.

**Table 1:** General characteristics of the sample.

	Placebo (*n* = 8)	β-alanine (*n* = 8)
Age (years)	30.3 ± 4.5	28.5 ± 3.2
Height (cm)	173 ± 0.1	172 ± 0.1
Weight (kg)	79.5 ± 11.2	73.4 ± 12.5
Body mass index (kg/m^2^)	25.6 ± 1.5	24.6 ± 2.2


### Procedure

#### Supplementation Protocol

β-Alanine and a placebo (resistant starch) were supplied for 23 days using a double-blinded method (Bex et al., 2014). The participants were randomly divided into two groups: placebo group (*n* = 8), ingested 6 g of resistant starch in capsules, divided into three times a day and β-alanine group (*n* = 8), ingested 5 g of β-alanine (GDS supplements—São Paulo, Brazil) and 1 g of resistant starch in capsules, divided into three times a day. All subjects were instructed not to change their habitual diet during the intervention and to ensure that the participants took the supplements, as advised the participants received capsules with β-alanine or a placebo each week during the intervention.

#### 10-km Running Test and Blood Lactate Concentration

All tests were conducted during the weekend on the same day and at the same hour. The 10-km running test was performed at baseline and after 23 days. Subjects performed a 5 min warm up and 5-min stretch and were informed about the running course and procedures. Time in the 10-km running test was measured and registered by a member of the research team who was waiting for the subjects at the end of the course. Subjects were instructed to wear the same kind of clothing (light shorts, light t-shirt, and running shoes) in every test. Tests were executed at the same time of the day, temperature, and humidity conditions, according to the CGE (official local weather forecast information). Blood lactate concentration was measured through the collection of a drop of blood from the fingertip on a reagent strip using a Roche portable lactate analyzer. The analyses were collected immediately after the 10-km running tests.

#### Running Training Protocol

All groups received a standard training program with duration of 23 days, three running sessions per week on non-consecutive days. On the first day of each week, subjects were instructed to run a moderate volume (7 km). On the second day of training, the participants performed six sprints of 500 m at maximum speed with a 2 min recovery interval between sprints. On the third of training, the volunteers ran a long distance (12 km). To ensure that the running training protocol was appropriate, all routine were supervised by researchers. When the participants ran a long distance, trained monitors were positioned each 1000 m across distance to better control.

### Statistical Analysis

A 2 × 2 (group × moment) repeated measures analysis of variance (RMANOVA) with the Bonferroni adjustment for multiple comparisons was used to compare lactate concentration and performance. For all measured variables, the estimated sphericity was verified according to the Mauchly’s *W* test and the Greenhouse–Geisser correction was used when necessary. The partial eta-squared (η^2^) was calculated for moment. The confidence intervals (CI-95%) was calculated and effect size for performance and lactate concentration was calculated via Cohen’s *d* [(treatment mean - placebo mean)/pooled SD] whereby a value of >0.20 was considered small, >0.50 moderate, and >0.80 large. Statistical significance was set at *p* < 0.05. The data were analyzed using Statistic software (version 10).

## Results

**Table 1** presents the mean and SD values for age, body weight, and height at baseline in the placebo and beta-alanine groups. There were no statistically significant differences between groups at baseline for any variable investigated.

**FIGURE 2:**
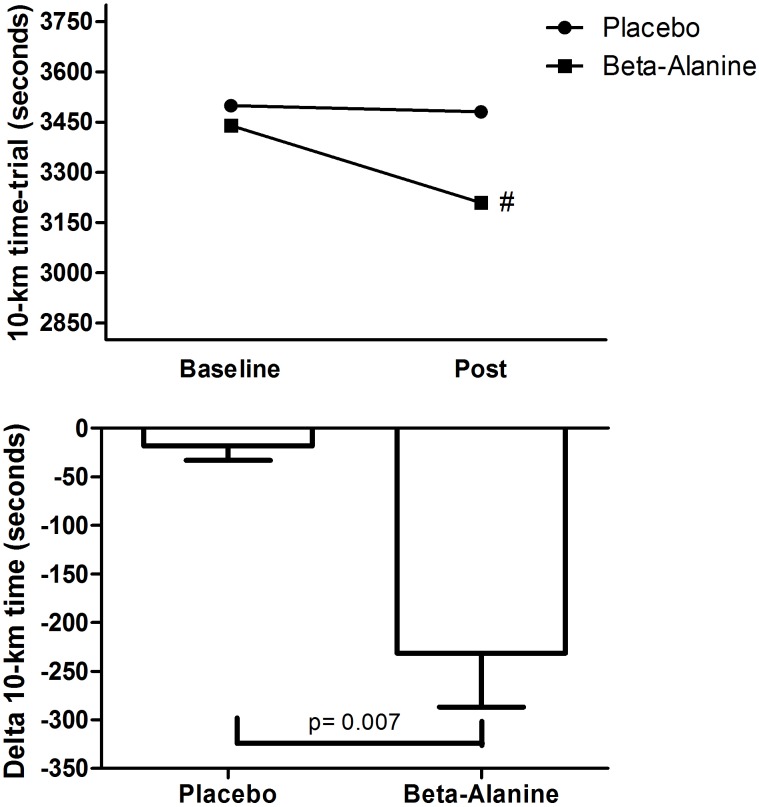
Comparison between placebo and beta-alanine group according to 10-km running performance. 1-A = Time to complete the 10-km at baseline and final (seconds); 1-B (Delta, time post-final minus time at the baseline value). #Bonferroni’s test with *p* < 0.05 compared to baseline.

**Figure 2** shows the differences in performance and delta for time between the placebo and β-alanine groups.

For performance (time in seconds), there was a main effect of time (*F* = 14.307, *p* = 0.003, η^2^ = 0.54) and a statistically significant interaction (*F* = 10.439, *p* = 0.007). The *post hoc* analysis revealed that time decreased significantly after 23 days only for the β-alanine group (*p* = 0.001), but no differences between groups were observed. However, when analyzing the delta (Time post-23 days minus Time at the baseline value) there was a statistically significant reduction for the β-alanine (*t* = 3.231, *p* = 0.007, CI-95% = 69.4-357.0) in relation to placebo group. Effect sizes were moderate for β-alanine group (0.78) and small for placebo group (0.05).

**FIGURE 3:**
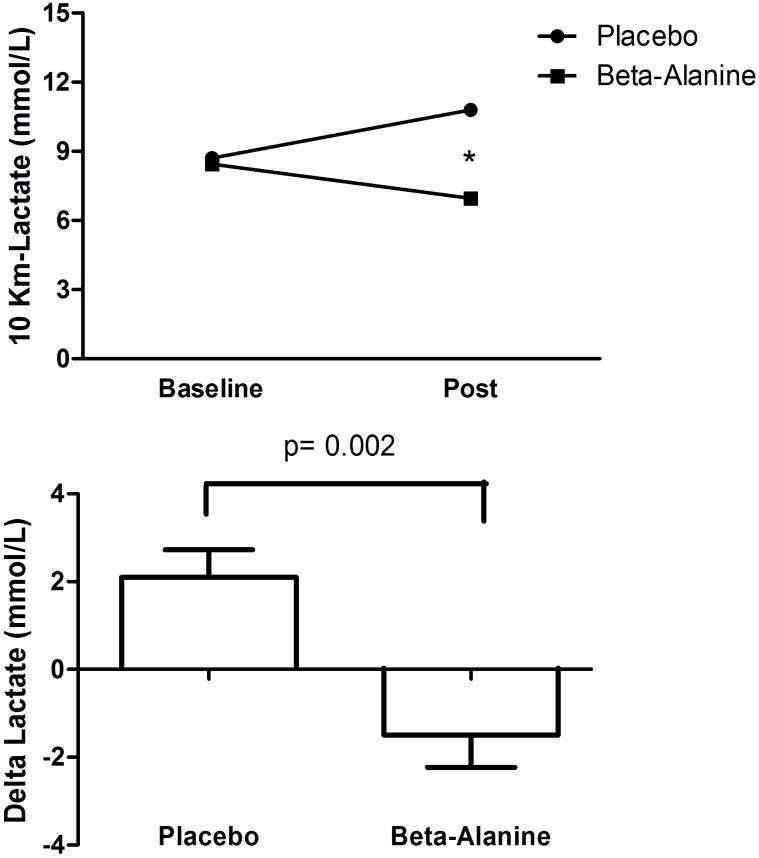
Comparison between placebo and beta-alanine group according to lactate concentration after 10 km running. 3-A = Lactate concentrations after 10-km at baseline and final (mmol/L); 3-B = Delta lactate concentrations after 10-km (post-final minus baseline value, mmol/L). ^∗^significant difference between group.

**Figure 3** presents the differences in the lactate concentration between the placebo and β-alanine groups.

For lactate, there was a significant interaction (*F* = 14.043, *p* = 0.002) with lower lactate concentration in the β-alanine group compared to the placebo (*p* = 0.036), however, there was no main effect of time (*F* = 0.390, *p* = 0.542, η^2^ = 0.54). The *post hoc* also showed that lactate was increased only in the placebo group (*p* = 0.047) but not in the β-alanine group (*p* = 0.266) after the 10-km running time trial. Furthermore, the delta was significantly lower for the β-alanine than placebo group (*t* = 3.747, *p* = 0.002, CI-95% = 1.5-5.7). Effect sizes were moderate for β-alanine group (0.68) and placebo group (0.77).

## Discussion

To our knowledge, this was the first study to investigate the effects of β-alanine supplementation on a 10-km running time trial in physically active adults. The main finding of this study was that β-alanine supplementation improved performance in 10-km after 23 days of supplementation, with lower lactate concentration.

A meta-analysis conducted by Hobson et al. (2012) demonstrated that the efficacy of β-alanine supplementation on exercise capacity is dependent on time and intramuscular H^+^ accumulation. They found that β-alanine supplementation was most effective in high-intensity exercise with a duration between 1 and 4 min, showing no effect of β-alanine supplementation in exercises shorter than 60 s. Another meta-analysis found similar results, in which β-alanine supplementation had greater impact in exercises with a duration between 0.5 and 10 min, however, the authors reported that there is a lack of research analyzing β-alanine in continuous exercises (Saunders et al., 2017), and the majority of these studies used an incremental protocol, demonstrating that β-alanine can enhance the total work done in exercise over 4 min (Stout et al., 2007; Smith et al., 2009), however, the effects of β-alanine on performance beyond 25 min running is unclear in the literature (Trexler et al., 2015).

Furthermore, the majority of investigations of β-alanine in the literature used a cycle ergometer, but few studies have analyzed the influence of β-alanine supplementation on running performance. Smith et al. (2009) showed that β-alanine supplementation (2 × 800 mg tablets, 3× daily) did not demonstrate improvement in 40 min of treadmill running in women. On the other hand, Ducker et al. (2013) analyzed the effects of β-alanine supplementation (4 weeks of 80 mg⋅kg-day) on 800 m track running performance in male recreational club runners and observed that race times were significantly faster following supplementation, decreasing by 3.64 s. In accordance with Ducker et al. (2013), we demonstrated improvement in performance, although during long-duration continuous exercises.

The ergogenic effect of β-alanine supplementation is widely due to the increase in intramuscular carnosine content, which improves skeletal muscle buffering capacity (Culbertson et al., 2010). Although long-distance running relies mainly on aerobic energy metabolism, some studies have demonstrated that mean running speed in prolonged running is dependent on lactate concentration, showing an association between lower lactate accumulations and higher running speed and anaerobic threshold (Sjodin and Jacobs, 1981; Fohrenbach et al., 1987; Tanaka, 1990). Our findings showed that β-alanine supplementation decreased lactate concentration after a 10-km running trial, suggesting that the improvement in performance was due in part to lower blood lactate accumulation.

Previous studies have investigated the influence of β-alanine on lactate accumulation during exercise. Glenn et al. (2015) found that β-alanine supplementation (four doses/day with 800 mg beta-alanine + 8 g dextrose) for 28 days increased by 23% a cycling test to exhaustion (equivalent to 120% maximal oxygen uptake) in female cyclists. In addition, they observed 24% lower lactate concentrations after the supplementation protocol. These findings corroborate with others (Jordan et al., 2010; Ghiasvand et al., 2012), showing that β-alanine can reduce blood lactate concentration during incremental running test. We hypothesize that the increase in 10-km running performance after β-alanine supplementation observed in the present study may be in part due to the increased muscular buffering capacity, mainly through lower demand on anaerobic glycolysis, generating lower lactate accumulation.

Furthermore, long running duration induced physiological and neuromuscular alterations that impair running speed (Davies and Thompson, 1986; Giandolini et al., 2016). There is a reduction in Ca^2+^ sensitivity and release from sarcoplasmic reticulum during prolonged running (Leppik et al., 2004), resulting in impaired interaction between actin-myosin filaments and lower force output (Homsher et al., 1996; Linari et al., 2015). Lower muscular excitability induced by prolonged running may be associated with the reduction in muscle glycogen and higher production of reactive oxygen species (ROS) (Duhamel et al., 2006; Gomez-Cabrera et al., 2006; Mrakic-Sposta et al., 2015). In this context, the increase in muscle carnosine induced by β-alanine supplementation attenuated fatigue, not only through its buffering capacities, but also its ability to improve myofibrillar Ca^2+^ release and sensitivity (Sale et al., 2010; Dutka et al., 2012). In addition, carnosine has also been reported to decrease ROS production, with an anti-oxidant activity (Kohen et al., 1988; Calabrese et al., 2005; Hipkiss, 2010). We hypothesize that the improvement in 10-km running performance induced by β-alanine supplementation in this study could also be explained by the effect of carnosine on intramuscular calcium influx and anti-oxidant activity, delaying neuromuscular fatigue. However, more studies are needed to better understand this mechanism.

Despite the importance of this study, some limitations need to be mentioned, such a lack of intramuscular analysis, muscle carnosine concentration, and muscle buffering capacity. Therefore, we suggest further research to analyze the effects of β-alanine supplementation on running time trials over different distances and investigate muscular adaptations in different populations, such as athletes.

In summary, β-alanine supplementation improved a 10-km running time trial and decreased blood lactate concentrations in physically active adults. These results suggest that β-alanine supplementation has positive effects on prolonged running.

## Clinical Implications

The present study suggests that β-alanine supplementation can be used as a nutritional strategy to improve performance in 10-km running by lowering blood lactate accumulation. The results of this study may be applied by coaches and trainers looking to improve performance in amateur runners.

## Author Contributions

EC devised the study design, participated in the interpretation of data, and drafted the manuscript. JS and DdS carried out the data collection, participated in the interpretation of data, and assisted in the writing of the manuscript. MdF, FL, and JR-N participated in the interpretation of data and drafted the manuscript. FR performed all statistical analysis, participated in the interpretation of data, and assisted in the writing of the manuscript. All authors read and approved the final manuscript.

## Conflict of Interest Statement

The authors declare that the research was conducted in the absence of any commercial or financial relationships that could be construed as a potential conflict of interest.
